# Supplementary material for: Identification of the genetic basis of pediatric neurogenetic disorders at a tertiary referral hospital in Indonesia: Contribution of whole exome sequencing
Source: PLoS One. 2023 Oct 25;18(10):e0293113. doi: 10.1371/journal.pone.0293113 (PMC10599538; doi:10.1371/journal.pone.0293113)
Supplement: S1 File — (DOCX) [file pone.0293113.s001.docx]

**S1 File. Schematics of Whole Exome Sequencing analysis workflow in 3billion**[1]

3billion performs exome capture with IDT xGen Exome Research Panel v2.0 (Integrated DNA Technologies, Coralville, Iowa, USA) and sequencing on NovaSeq 6000 (Illumina, San Diego, CA, USA). The IDT panel was selected after a thorough evaluation of the coverage statistics in comparison with other commercially available capture kits. Currently, the minimum depth-of-coverage (DOC) per exome is 100 x with a minimum 98% of the targeted region covered at 20x DOC.

Once the sequencing is complete, the base call (BCL) sequence files generated by NovaSeq 6000 are converted and demultiplexed to FASTQ files using bcl2fastq v2.20.0.422 [2]. Sequence reads in the FASTQ files are aligned to the human reference genome (GRCh37/hg19 from NCBI, February 2009) using BWA-mem 0.7.17 [3] to generate BAM files. BAM files are processed following the GATK best practices (GATK v.3.8) [4] for single nucleotide variants (SNV) and small insertions/deletions (indel) variant calling to generate VCF files [5], [6]. Conifer [7] and 3bCNV are used for copy number variant (CNV) calling based on depth-of-coverage (DOC) data. Due to the lack of sequencing data between exons, the resolution of CNV calls is minimum 3 consecutive exons and for most of the CNVs, exact breakpoints are not identifiable. AutoMap v1.2 [8] is used for Region of Homozygosity (ROH) detection from the VCF file (Figure attached). Various quality control metrics such as Q30, mapping rate, PCR duplication rate, capture efficiency, total number of variants, heterozygous/homozygous (het/homo), and transition/transversion (ts/tv) ratios are used to ensure the sequencing data is within an acceptable range for a clinical test.


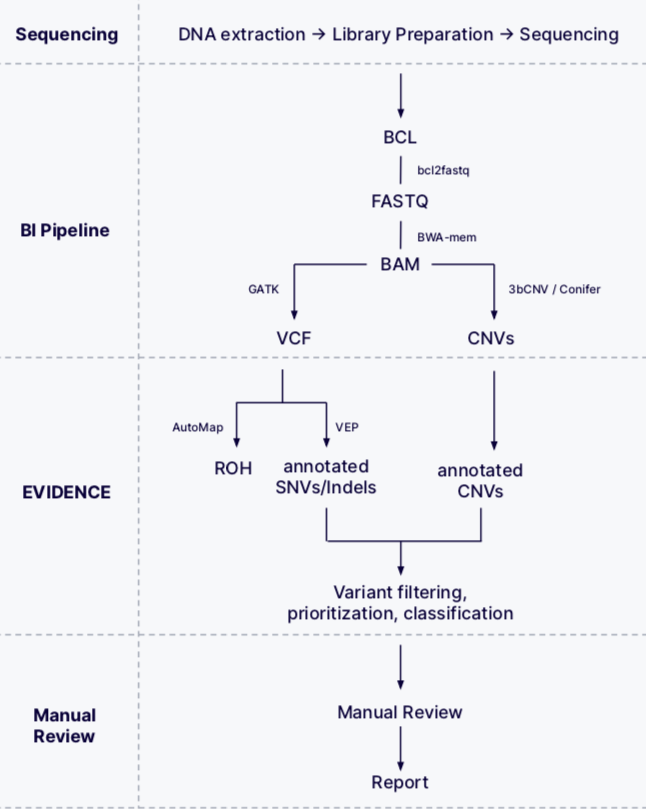


**Abbreviations**

BI: Bioinformatics

BCL: Base call

CNV: Copy number variation

GATK: Genome Analysis Toolkit

ROH: Region of homozygosity

SNV: Single nucleotide variants

VEP: Variant effect predictor

**Reference:**

[1] “3billion-White-Paper-2023_2023-03-23-010953_twyj.pdf.” Accessed: Sep. 25, 2023. [Online]. Available: https://3billion.io/img/common/3billion-White-Paper-2023_2023-03-23-010953_twyj.pdf

[2] “bcl2fastq Conversion Software.” Accessed: Sep. 25, 2023. [Online]. Available: https://support.illumina.com/sequencing/sequencing_software/bcl2fastq-conversion-software.html

[3] H. Li, “Aligning sequence reads, clone sequences and assembly contigs with BWA-MEM.” arXiv, May 26, 2013. doi: 10.48550/arXiv.1303.3997.

[4] “Best Practices Workflows – GATK.” Accessed: Sep. 25, 2023. [Online]. Available: https://gatk.broadinstitute.org/hc/en-us/sections/360007226651-Best-Practices-Workflows

[5] A. McKenna *et al.*, “The Genome Analysis Toolkit: A MapReduce framework for analyzing next-generation DNA sequencing data,” *Genome Res*, vol. 20, no. 9, pp. 1297–1303, Sep. 2010, doi: 10.1101/gr.107524.110.

[6] M. A. DePristo *et al.*, “A framework for variation discovery and genotyping using next-generation DNA sequencing data,” *Nat Genet*, vol. 43, no. 5, pp. 491–498, May 2011, doi: 10.1038/ng.806.

[7] N. Krumm *et al.*, “Copy number variation detection and genotyping from exome sequence data,” *Genome Research*, vol. 22, no. 8, p. 1525, Aug. 2012, doi: 10.1101/gr.138115.112.

[8] M. Quinodoz *et al.*, “AutoMap is a high performance homozygosity mapping tool using next-generation sequencing data,” *Nat Commun*, vol. 12, p. 518, Jan. 2021, doi: 10.1038/s41467-020-20584-4.
