# Supplementary material for: Identification of the genetic basis of pediatric neurogenetic disorders at a tertiary referral hospital in Indonesia: Contribution of whole exome sequencing
Source: PLoS One. 2023 Oct 25;18(10):e0293113. doi: 10.1371/journal.pone.0293113 (PMC10599538; doi:10.1371/journal.pone.0293113)
Supplement: Attachment — Submitted filename: ARN-PONE-D-23-14709.pdf [file pone.0293113.s002.pdf]

PLOS ONE

Identification of the genetic basis of pediatric neurogenetic disorders at a tertiary referral hospital in Indonesia: contribution of whole exome sequencing

--Manuscript Draft--

**Manuscript Number:**	PONE-D-23-14709
**Article Type:**	Research Article
**Full Title:**	Identification of the genetic basis of pediatric neurogenetic disorders at a tertiary referral hospital in Indonesia: contribution of whole exome sequencing
**Short Title:**	Identification of the genetic basis of pediatric neurogenetic disorders
**Corresponding Author:**	Elisabeth Siti Herini, MD (Paediatrician), PhD<br>Gadjah Mada University Faculty of Medicine: Universitas Gadjah Mada Fakultas Kedokteran Kesehatan Masyarakat dan Keperawatan<br>Yogyakarta, Daerah Istimewa Yogyakarta INDONESIA
**Keywords:**	Neurogenetic Disorders; Whole Exome Sequencing; Genetic etiology; Children; Indonesia
**Abstract:**	Background: Neurogenetic disorders (NGDs) are complex Mendelian disorders that affect the neurological system. A molecular diagnosis will provide more information about pathophysiology, prognosis, and therapy, including future genetic therapy options. Whole-Exome Sequencing (WES) can rapidly discover the genetic basis in NGDs.<br>Objective: The purpose of this study was to assess the WES results and its value in diagnosing pediatric NGDs, especially those with unspecified clinical features.<br>Methods: A retrospective chart review was performed from May 2021- May 2022 in Dr. Sardjito General Hospital, a tertiary referral hospital in Yogyakarta, Indonesia. WES proband only was conducted on children aged 0 to 17 years old who met one or more of the following criteria: (1) epileptic encephalopathy and familial epilepsy; (2) complex neurodevelopmental phenotypes; (3) leukodystrophy; (4) movement disorders; and (5) neurocutaneous disorder. The WES was conducted in the certified laboratory, 3Billion, in Seoul, Korea.<br>Results: The diagnosis yield of WES in our study was 45% (9/20). We identified nine positive results, including eight pathogenic single nucleotide variants (SNVs) in 8 genes (KCNQ2, ARSA, UBE3A, IRF2BPL, ATM, MECP2, TSC2, and NF1), and one variant with uncertain significance (VUS) in the ADKgene that has not been able to explain the observed clinical features. Of the nine patients with positive WES results, five had missense mutations, three frameshift mutations, and one nonsense mutation. Additionally, we identified two suggestive copy number variants (CNVs) in 15q11.2q13.1and 1p31.3.<br>Conclusions: Whole-Exome Sequencing is an essential diagnostic tool for pediatric NGDs, especially those with unspecified clinical features. It ends multi-year diagnostic odysseys, provides personalized medicine therapy, and optimizes genetic counselling for these families.
**Order of Authors:**	Agung Triono, MD (Pediatric Neurologist)
	Elisabeth Siti Herini, MD (Pediatric Neurologist), PhD
	Kristy Iskandar, MD(Pediatric Neurologist), Ph.D
	Marissa Leviani Hadiyanto, MD
	Andika Priamas Nugrahanto, MD
	Kania Diantika, MD
	Veronica Wulan Wijayanti, MD
**Additional Information:**	
**Question**	**Response**
**Financial Disclosure**	This study was supported by grants from the Ministry of Research and

Powered by Editorial Manager® and ProduXion Manager® from Aries Systems Corporation

Enter a financial disclosure statement that describes the sources of funding for the work included in this submission. Review the submission guidelines for detailed requirements. View published research articles from *PLOS ONE* for specific examples.<br><br>This statement is required for submission and **will appear in the published article** if the submission is accepted. Please make sure it is accurate.<br><br>**Unfunded studies**<br>Enter: *The author(s) received no specific funding for this work.*<br><br>**Funded studies**<br>Enter a statement with the following details:<br>• Initials of the authors who received each award<br>• Grant numbers awarded to each author<br>• The full name of each funder<br>• URL of each funder website<br>• Did the sponsors or funders play any role in the study design, data collection and analysis, decision to publish, or preparation of the manuscript?<br>• **NO** - Include this sentence at the end of your statement: *The funders had no role in study design, data collection and analysis, decision to publish, or preparation of the manuscript.*<br>• **YES** - Specify the role(s) played.<br><br>* typeset	Technology/National Research and Innovation Agency of the Republic of Indonesia to AT under contract numbers 018/E5/PG.02.00.PT/2022 and 1633/UN1/DITLIT/Dit-Lit/PT.01.00.2022 T. The funders had no role in study design, data collection and analysis, decision to publish, or preparation of the manuscript.<br><br>Url of funder website:<br>https://bima.kemdikbud.go.id
**Competing Interests**<br><br>Use the instructions below to enter a competing interest statement for this submission. On behalf of all authors, disclose any competing interests that could be perceived to bias this work—acknowledging all financial support and any other relevant financial or non-financial competing interests.<br><br>This statement is required for submission and **will appear in the published article** if	The authors have declared that no competing interests exist.

the submission is accepted. Please make sure it is accurate and that any funding sources listed in your Funding Information later in the submission form are also declared in your Financial Disclosure statement.<br><br>View published research articles from *PLOS ONE* for specific examples.<br><br>**NO authors have competing interests**<br><br>Enter: *The authors have declared that no competing interests exist.*<br><br>**Authors with competing interests**<br><br>Enter competing interest details beginning with this statement:<br><br>*I have read the journal's policy and the authors of this manuscript have the following competing interests: [insert competing interests here]*<br><br>* typeset	
**Ethics Statement**<br><br>Enter an ethics statement for this submission. This statement is required if the study involved:<br><br>• Human participants<br>• Human specimens or tissue<br>• Vertebrate animals or cephalopods<br>• Vertebrate embryos or tissues<br>• Field research<br><br>Write "N/A" if the submission does not require an ethics statement.<br><br>General guidance is provided below. Consult the submission guidelines for detailed instructions. **Make sure that all information entered here is included in the Methods section of the manuscript.**	The Medical and Health Research Ethics Committee approved all recruitment and research protocol of the Faculty of Medicine, Public Health and Nursing, Universitas Gadjah Mada (Ethical Clearance Number: K.E./0550/06/2020; Dated June 2020). Patients older than 12 years old and/ or their parents or guardian (for patients

**Format for specific study types**<br><br>**Human Subject Research (involving human participants and/or tissue)**<br>• Give the name of the institutional review board or ethics committee that approved the study<br>• Include the approval number and/or a statement indicating approval of this research<br>• Indicate the form of consent obtained (written/oral) or the reason that consent was not obtained (e.g. the data were analyzed anonymously)<br><br>**Animal Research (involving vertebrate animals, embryos or tissues)**<br>• Provide the name of the Institutional Animal Care and Use Committee (IACUC) or other relevant ethics board that reviewed the study protocol, and indicate whether they approved this research or granted a formal waiver of ethical approval<br>• Include an approval number if one was obtained<br>• If the study involved *non-human primates*, add *additional details* about animal welfare and steps taken to ameliorate suffering<br>• If anesthesia, euthanasia, or any kind of animal sacrifice is part of the study, include briefly which substances and/or methods were applied<br><br>**Field Research**<br><br>Include the following details if this study involves the collection of plant, animal, or other materials from a natural setting:<br>• Field permit number<br>• Name of the institution or relevant body that granted permission	
**Data Availability**<br><br>Authors are required to make all data underlying the findings described fully available, without restriction, and from the time of publication. PLOS allows rare exceptions to address legal and ethical concerns. See the PLOS Data Policy and FAQ for detailed information.	Yes - all data are fully available without restriction

A Data Availability Statement describing where the data can be found is required at submission. Your answers to this question constitute the Data Availability Statement and **will be published in the article**, if accepted.<br><br>**Important:** Stating 'data available on request from the author' is not sufficient. If your data are only available upon request, select 'No' for the first question and explain your exceptional situation in the text box.<br><br>Do the authors confirm that all data underlying the findings described in their manuscript are fully available without restriction?	
**Describe where the data may be found in full sentences. If you are copying our sample text, replace any instances of XXX with the appropriate details.**<br><br>• If the data are **held or will be held in a public repository**, include URLs, accession numbers or DOIs. If this information will only be available after acceptance, indicate this by ticking the box below. For example: *All XXX files are available from the XXX database (accession number(s) XXX, XXX.*).<br>• If the data are all contained **within the manuscript and/or Supporting Information files**, enter the following: *All relevant data are within the manuscript and its Supporting Information files.*<br>• If neither of these applies but you are able to provide **details of access elsewhere**, with or without limitations, please do so. For example:<br><br>*Data cannot be shared publicly because of [XXX]. Data are available from the XXX Institutional Data Access / Ethics Committee (contact via XXX) for researchers who meet the criteria for access to confidential data.*<br><br>*The data underlying the results presented in the study are available from (include the name of the third party*	All relevant data are within the manuscript and its Supporting Information files.

and contact information or URL).<br>• This text is appropriate if the data are owned by a third party and authors do not have permission to share the data.<br><br>* typeset	
Additional data availability information:	

 Click here to access/download;Manuscript;main manuscript.docx

Full Title:

Identification of the genetic basis of pediatric neurogenetic disorders at a tertiary referral hospital in Indonesia: contribution of whole exome sequencing

Short Title:

Identification of the genetic basis of pediatric neurogenetic disorders

Agung Triono[1], Kristy Iskandar[2], Marissa Leviani Hadiyanto[1], Andika Priamas Nugrahanto[1], Kania Diantika[1], Veronica Wulan Wijayanti[1], Elisabeth Siti Herini[1*]

[1]Department of Child Health, Faculty of Medicine, Public Health and Nursing, Universitas Gadjah Mada, Dr. Sardjito Hospital, Yogyakarta 55281, Indonesia

[2]Department of Child Health, Faculty of Medicine, Public Health and Nursing, Universitas Gadjah Mada, Academic UGM Hospital, Yogyakarta 55291, Indonesia

*** Corresponding author**

Elisabeth Siti Herini, MD, PhD, Professor

Department of Child Health

Faculty of Medicine, Public Health and Nursing, Universitas Gadjah Mada

Dr. Sardjito Hospital

Jl. Kesehatan No. 1 Yogyakarta 55281, Indonesia

Tel/Fax: +62-274-561616/ +62-274-583745

E-mail: herini_es@ugm.ac.id

ABSTRACT

Background: Neurogenetic disorders (NGDs) are complex Mendelian disorders that affect the neurological system. A molecular diagnosis will provide more information about pathophysiology, prognosis, and therapy, including future genetic therapy options. Whole-Exome Sequencing (WES) can rapidly discover the genetic basis in NGDs.

Objective: The purpose of this study was to assess the WES results and its value in diagnosing pediatric NGDs, especially those with unspecified clinical features.

Methods: A retrospective chart review was performed from May 2021- May 2022 in Dr. Sardjito General Hospital, a tertiary referral hospital in Yogyakarta, Indonesia. WES proband only was conducted on children aged 0 to 17 years old who met one or more of the following criteria: (1) epileptic encephalopathy and familial epilepsy; (2) complex neurodevelopmental phenotypes; (3) leukodystrophy; (4) movement disorders; and (5) neurocutaneous disorder. The WES was conducted in the certified laboratory, 3Billion, in Seoul, Korea.

Results: The diagnosis yield of WES in our study was 45% (9/20). We identified nine positive results, including eight pathogenic single nucleotide variants (SNVs) in 8 genes (*KCNQ2*, *ARSA*, *UBE3A*, *IRF2BPL*, *ATM*, *MECP2*, *TSC2*, and *NF1*), and one variant with uncertain significance (VUS) in the *ADK* gene that has not been able to explain the observed clinical features. Of the nine patients with positive WES results, five had missense mutations, three frameshift mutations, and one nonsense mutation. Additionally, we identified two suggestive copy number variants (CNVs) in *15q11.2q13.1* and *1p31.3*.

Conclusions: Whole-Exome Sequencing is an essential diagnostic tool for pediatric NGDs, especially those with unspecified clinical features. It ends multi-year diagnostic odysseys, provides personalized medicine therapy, and optimizes genetic counselling for these families.

Keywords: **Neurogenetic Disorders, Whole Exome Sequencing, Genetic etiology, Children, Indonesia**

INTRODUCTION

Neurogenetic disorders (NGDs) are a complex group of Mendelian disorders that affect the nervous system and have a variety of clinical manifestations [1,2]. More than half of all genetic anomalies impact the central and/or peripheral nerve systems, either alone or in conjunction with other systems, and the majority of these disorders manifest in childhood [3]. This genetic etiology is especially prevalent in neurologic disorders, such as that causing epilepsy, ataxia, developmental delay, special myopathy, movement disorders, intellectual disability, and others [4]. Many NGDs manifest in early childhood, resulting in early death or lifelong disability. The prevalence of NGDs is about 1 in 1,100 of the general population who experience neurological disorders with a single gene [5]. Mutations of a gene can lead to different phenotypes, whereas several other genes can cause the same phenotype [4]. Furthermore, these diseases represent an enormous burden on the affected individuals, including families and communities [6].

The science of pediatric neurogenetics has rapidly developed in the last decade. The development of molecular diagnostic technology reports gene mutations responsible for neurological disorders from various parts of the world [3]. Obtaining a specific molecular diagnosis will provide additional information regarding the pathophysiology, prognosis, and therapy, including genetic therapy opportunities in the future [7]. Next-generation sequencing (NGS) is a new DNA sequencing technique that can sequence a large number of genes, including targeted panel sequencing, whole-exome sequencing (WES) and whole-genome sequencing (WGS). NGS has the capacity to detect several mutations, including de novo, novel, and familial variants [8]. WES focuses on protein-coding sections of the genome, which make up about 1% to 2% of the genome yet contain 85% of mutations with a substantial influence on phenotype [9].

The application of WES technology in research and diagnostic facilities has resulted in the quick discovery of the genetic basis in NGDs. Establishing a genetic etiology in NGDs can also be useful in future planning for the patient and family since it can provide information on prognosis and gene-specific medicines, "precision medicine" [10]. To the best of our knowledge, no research has ever been conducted in our country on the use of WES examination for the basic molecular diagnosis of NGDs. We conducted a retrospective chart review of 20 unrelated individuals with NGDs to assess the WES examination as a diagnostic tool for pediatric NGDs, especially those with unspecified clinical features in routine practice by a tertiary referral hospital in a developing country. All participants had undergone several clinical evaluations and diagnostic tests over the years.

METHODS

Clinical samples

A retrospective chart review of 20 children with NGDs was performed in the Neuropediatric Clinic at a tertiary regional hospital in Yogyakarta, Indonesia, from May 2021 to May 2022. These patients were chosen as participants for this study based on the presence of NGD symptoms with ages between 0 and 17 years. Inclusion criteria were: (1) Epileptic encephalopathy and familial epilepsy; (2) Complex neurodevelopmental phenotypes, such as microcephaly, and macrocephaly, with unique features of movement disorder and/or epilepsy; (3) Leukodystrophy; (4) Movement disorders, includes familial ataxia, paroxysmal dyskinesia, and/or choreoathetosis with a history of cerebral palsy or epilepsy, early onset dystonia, familial myoclonus, complex movement disorder; and (5) Neurocutaneous disorders. Patients were included if the pediatric neurologists had a strong suspicion that the patient had a genetic disease that met the inclusion

criteria. We collected detailed demographic, perinatal and family history, disease progression characteristics, developmental status, comorbidities, and relevant clinical testing such as brain magnetic resonance imaging (MRI), and electroencephalography (EEG). Experienced pediatric neurologists assessed the clinical seizure semiology, EEG results, and brain imaging reports of the patients. All patients' exomes were sequenced and analyzed as probands after getting the patients' or guardians' informed consent. According to the American College of Medical Genetics (ACMG) guidelines (v2.0) [11], patients and/or their legal guardians were informed of the potential revelation of secondary medical outcomes and offered the opportunity to receive the data. If the proband's WES results were positive and the parents were willing to search for the genetic disorder's inheritance, the examination continued to Sanger sequencing of the parents and siblings. The WES and Sanger sequencing were performed in an accredited laboratory, 3Billion, Seoul, Korea. This study was approved by the Medical and Health Research Ethics Committee of the Faculty of Medicine, Public Health and Nursing at Universitas Gadjah Mada, Yogyakarta, Indonesia (K.E./0550/06/2020).

Whole exome sequencing

Genomic DNA was extracted from each patient's blood samples. The xGen Exome Research Panel v2 was used for exome capture, and the NovaSeq 6000 was used for sequencing. About 99.0% of the targeted bases had a depth of 20x. A total of 69.,490 single nucleotide variants (SNV) were identified, and 11,951 small insertions and deletions (indel). On request, the entire variant list is available without annotation or clinical interpretation. Despite insufficient coverage across 1% of the bases, these metrics are consistent with high-quality exome sequencing data and are deemed sufficient for analysis.

Variant interpretation

Variant interpretation was performed using EVIDENCE [12], an in-house software developed to prioritize variants based on the guidelines recommended by the ACMG and the AMP [13] in the context of the patient's phenotype, relevant family history and previous test results provided by the ordering physician [11]. The databases that are automatically updated consist of public databases, in-house variant databases, and manually curated literature databases. At the time of variant interpretation, only variants deemed clinically significant and relevant to the patient's primary clinical indications will be reported. Parental and sibling samples (when available) were also tested using Sanger sequencing to confirm clinically relevant variants with proband. The results are interpreted based on the ACMG and AMP standards utilizing a five-tier terminology system called "**pathogenic**," "likely pathogenic," "**variant uncertain significance (VUS)**," "likely benign," and "benign" to describe variants identified in genes that cause Mendelian disorders [13].

RESULTS

Characteristics of the patients

Twelve males and eight females were included in the study, with the age of presentation ranging from 0 to 17 years. The mean age at onset was 2 .45 years ± 3.23 years and the mean age at genetic testing was 6.57 years ± 5.47 years. Table 1 summarizes the demographic and clinical information of the patients. All patients had various neurological manifestations such as: intractable seizure, developmental delay, intellectual delay, regression of milestones, facial dysmorphic, visual impairment, hearing abnormalities, abnormal movement, abnormal gait, abnormal neuroimaging and EEG. Eight patients had primary features of developmental and epileptic encephalopathy (DEE), two patients with leukodystrophy, four patients with a complex

neurodevelopmental phenotype, four patients with movement disorders, and two with neurocutaneous disorders, based on the phenotypic evaluation of the pediatric neurologist.

Brain MRI and/or computerized tomography (CT) scans were done on almost all patients (16 out of 20 patients), while the Electroencephalogram (EEG), Electroneuromyography (ENMG) and Electromyography (EMG) were performed according to medical indications. The most common neuroradiological findings were cerebral atrophy (45%), ventriculomegaly (30%), hippocampal atrophy (15%), and hydrocephalus (10%). EEG was performed on 12 of 20 patients, and most had epileptiform discharge with abnormal diffuse irritative.

Table 1. The demographic and clinical features of patients selected for WES

Patient ID (#)	Primary disease classification	Gender	Familial recurrence	Age at onset	Age at testing	Clinical manifestation	Neuroimaging findings	Other neuroradiologic test
#1	DEE	M	-	1 day	2 years	Intractable seizure, global developmental delay, cerebral palsy, spastic tetraparesis	Cerebral atrophy, ventriculomegaly, and subdural hygroma	EEG: Abnormal epileptiform
#2	DEE	M	-	4 years	15 years	intractable seizure, intellectual disability, Generalized tonic clonic, lower extremity hypertonia, stereotypic movement (clapping hands and head), history of prematurity and low birth weight	Cerebral atrophy, hydrocephalus communicans, hippocampal atrophy, ventriculomegaly	EEG: abnormally irritating with diffuse epileptiform discharge
#3	DEE	M	History of twin with the same symptoms who died.	2 years	7 years	intractable seizure, generalized tonic-clonic seizures, cerebral palsy, spastic tetraparesis, speech delay, severe neural hearing loss	N/A	EEG: epileptiform discharge with burst suppression
#4	DEE	M	-	5 years	12 years	intractable seizure, global developmental delay generalized tonic-clonic seizures, neurodevelopmental regression, intellectual disability	Cerebral edema, microcalcifications, and cerebritis	EEG: epileptiform discharge with abnormal diffuse irritative
#5	DEE	M	-	2 day	1 year	intractable seizure, global developmental delay, focal seizures, strabismus, congenital Hypothyroid, Hypermetropia, cerebral atrophy, infantile spasm	Cerebral atrophy, hippocampal atrophy	EEG: epileptiform discharge with abnormal diffuse irritative and burst suppression
#6	DEE	M	-	0 day	0.5 year	Intractable seizure, global developmental delay, hypertonia, Dysmorphic facies	Lissencephaly, Microcephaly, Ventriculomegaly, Bilateral enlargement of the subarachnoid space in the frontotemporoparietal region	N/A
#7	DEE	M	-	3 years	8 years	speech delay, generalized tonic-clonic seizures, absence seizure	N/A	EEG: Abnormal epileptiform
#8	DEE	F	-	1 day	1.5 years	Infantile spasm, intractable seizure, cerebral atrophy, microcephaly	microcephaly with cerebral atrophy and ventriculomegaly	EEG: epileptiform discharge with abnormal diffuse irritative
#9	leukodystrophy	F	-	1.5 years	4 years	Regression of development, generalized tonic clonic seizure and spasticity	white matter changes extensive bihemisphere cerebri	N/A
#10	leukodystrophy	F	-	4 day	2 years	Intractable seizure, Focal tonic seizure, global developmental delay, laryngomalacia	Cerebral atrophy, ventriculomegaly, leukodystrophy	EEG: epileptiform discharge with abnormal diffuse irritative
#11	complex neurodevelopmental	M	-	0 day	0.5 year	macrocephaly, hypotonia generalisata and facial dysmorphic in the form of frontal bossing, wide forehead, drooping ears, high palatal arch, micrognathia	Cerebral atrophy, ventriculomegaly, and hypoplasia of the corpus callosum	N/A
#12	complex neurodevelopmental	M	-	0 day	2 years	Coloboma palpebra, nevus sebaceous, infantile spasm, Generalized tonic clonic, intractable seizure, sensorineural hearing loss, global developmental delay	Cerebral atrophy, hippocampal atrophy, communicating hydrocephalus and periventricular cyst	EEG: modified hypsarrhythmia
#13	complex neurodevelopmental	F	History of brother with CP, and sister with speech delay	3 months	5 years	Speech delay, Attention Deficit Hyperactivity Disorder	N/A	N/A
#14	complex neurodevelopmental	F	-	6 months	4 years	movement and balance disorder, frequent smiling, apparent happy demeanor, speech impairment, absence of seizure	microcephaly, corpus callosum dysgenesis, and heterotopia grey matter on the bilateral lateral ventricle	EEG: abnormal pattern during sleep with medium amplitude rhythmic 2-3c/s
#15	movement disorder	M	-	5 years	10 years	Progressive ataxia, Drooling, Slurred speech, Muscle flaccidity	Pathological hyperintensity on FLAIR sequence in bilateral frontal lobes, bilateral basal ganglia, and hippocampus, particularly the left hippocampus.	EMG: myopathy<br>ENMG: motor and sensory results were within normal limits.

#16	movement disorder	F	History of older sister with movement disorder	6 years	9 year	Difficulty walking, dysarthria, telangiectasia	Cerebellum atrophy	N/A
#17	movement disorder	M	-	10 years	12 years	Dextroscoliosis thoracolumbalis, uncontrolled movement, microcephaly	Bilateral temporal lobe atrophy	N/A
#18	movement disorder	F	-	2 years	3 years	Global developmental delay, microcephaly	N/A	N/A
#19	neurocutaneous disorder	F	-	10 years	17 years	absence and focal epilepsy, borderline intellectual functioning, organic psychosis, multiple hypomelanotic maculae, angiofibroma, and shagreen patch	Cerebral atrophy, cortical and subcortical tubers in the parietal and occipital lobes	EEG: diffuse epileptiform abnormalities with focus on the left hemisphere
#20	neurocutaneous disorder	M	-	3 months	16 years	Generalized tonic clonic seizure, tetraparese spastic, microcephaly cafe au lait spots, Freckling in the armpits or groin area, Lisch nodules, neurofibromas	Cerebral atrophy	EEG: hypsarrthymia, generalizes periodic epileptiform discharge

DEE: developmental and epileptic encephalopathy; EEG: Electroencephalogram; ENMG: Electroneuromyography; EMG: Electromyography

Exome Sequencing result

Overall, the diagnosis yield in our study was 45% (9/20) (Fig 1). We identified nine positive results, including eight pathogenic single nucleotide variants (SNVs) in 8 genes (*KCNQ2*, *ARSA*, *UBE3A*, *IRF2BPL*, *ATM*, *MECP*, *TSC2*, and *NF1*), one VUS SNVs in the *ADK* gene that has not been able to explain the observed clinical characteristics (Picture 1). All of them are variants that have been published before. Classifications for pathogenic and VUS WES-positive results were identified, but no likely pathogenic, benign, or likely benign variants were identified in this study. Of the 9 patients with positive WES results, 5 had missense mutations, 3 frameshift mutations, and 1 nonsense mutation. Additionally, we identified two suggestive copy number variants (CNVs) in 15q11.2q13.1 and 1p31.3. In patients with suggestive CNV results, we have not continued further examinations such as chromosomal microarray (CMA). The remaining 11 patients in our study had negative WES results. The average age at onset in the WES-positive group was 3.37 years± 3.36 years, and age at genetic testing was 8.55 years ± 5.65 years. The detailed information about genetic findings of our patients are summarized in Table 2.

(insert figure 1 here)

Figure 1. Whole-Exome Sequencing Result.

Table 2. Summary of patients with established molecular diagnosis by WES.

Patient ID- (sex)	Primary disease classification	Variant interpretation	Family history	Phenotype	Genes	genomic position (GRCh37)	cDNA changes	Protein changes	Name of disease (OMIM)	Zygosity (Inheritance)
#1-M	DEE	Pathogenic		Intractable seizure, global developmental delay, cerebral palsy, spastic tetraparesis	*KCNQ2*	20-62071010-C-T	NM_172107.4:c.868G>A (missense mutation)	NP_742105.1:p.Gly290Ser	Epileptic encephalopathy, early infantile, (613720)	Heterozygous (unknown)
#9-F	leukodystrophy	Pathogenic		Regression of development, generalized tonic clonic seizure and spasticity	*ARSA*	22-51065363-A-AG	NM_000487.6:c.582dup (frameshift mutation)	NP_000478.3:p.Trp195LeufsTer15	Metachromatic leukodystrophy (250100)	Heterozygous (unknown)
#14- F	complex neurodevelopmental	Pathogenic	+	Movement and balance disorder, frequent smiling, apparent happy demeanor, speech impairment, absence seizure	*UBE3A*	15-25615817-G-A	NM_000462.5:c.1513C>T (frameshift mutation)	NP_000453.2:p.Arg505Ter	Angelman Syndrome (105830)	Heterozygous (unknown)
#15- M	movement disorder	Pathogenic		Progressive ataxia, Drooling, Slurred speech, Muscle flaccidity	*IRF2BPL*	14-77493637-G-A	NM_024496.4:c.499C>T (missense mutation)	NP_078772.1:p.Gln167Ter	Neurodevelopmental disorder with regression, abnormal movements, loss of speech, and seizures (618088)	Heterozygous (unknown)
#16- F	movement disorder	Pathogenic	+	Ataxia, dysarthria, telangiectasia	*ATM*	11-108214053-C-A	NM_000051.4:c.8373C>A (stop gained-nonsense mutation)	NP_000042.3:p.Tyr2791Ter	Ataxia-telangiectasia syndrome (208900)	Homozygous (autosomal recessive)
#18- F	movement disorder	Pathogenic		Global developmental delay, microcephaly	*MECP2*	X-153296363-G-A	NM_001110792.2:c.952C>T (missense mutation)	NP_001104262.1:p.Arg318Cys	X-linked Rett syndrome (OMIM: 312750)	Heterozygous (unknown)
#19- F	neurocutaneous disorder	Pathogenic		Absence and focal epilepsy, borderline intellectual functioning, organic psychosis, multiple hypomelanotic maculae, angiofibroma, and shagreen patch	*TSC2*	16-2137898-C-T	NM_000548.5:c.5024C>T (missense mutation)	NP_000539.2:p.Pro1675Leu	Tuberous Sclerosis 2 (61325)	Heterozygous (de novo)

#20- M	neurocutaneous disorder	Pathogenic	Generalized tonic clonic seizure, tetraparese spastic, microcephaly, cafe au lait spots, Freckling, Lisch nodules, neurofibromas	*NF1*	17-29576097-TC-T	NM_001042492.3:c.4076del (frameshift mutation)	(NP_001035957.1:p.Pro1359LeufsTer19	Neurofibromatosis, type 1 (162200)	Heterozygous (de novo)
#4- M	DEE	VUS	Intractable seizure, global developmental delay, generalized tonic-clonic seizures, neurodevelopmental regression, intellectual disability	*ADK*	10-76153999-T-C	NM_006721.4:c.374T>C (missense mutation)	NP_006712.2:p.Val125Ala	Hypermethioninemia due to adenosine kinase deficiency (614300)	Homozygous (unknown)

DEE: developmental and epileptic encephalopathy; VUS: variant uncertain significance.

Parental Sanger sequencing was performed on three patients with pathogenic SNV (patients #16, #19, and #20) to determine inheritance patterns. As a result, two out of three SNV abnormalities were caused by de novo mutations, as evidenced by negative parental Sanger results. Meanwhile, patient #16 had autosomal recessive inheritance which was inherited from asymptomatic parents. Sanger sequencing revealed that the patient (II-2), her sibling (II-1) and her parents (I-1 and I-2) carried the compound heterozygotes of a known nonsense mutation c.8373C>A (p. Tyr2791Ter) in the ATM gene (Fig 2). The patient's 16-year-old brother displayed all the symptoms of ataxia but with a more severe clinical progression. He normally developed until the age of eight, and the symptoms of typical ataxia appeared at nine years old. Both eyes had severe conjunctival hyperemia, with difficulty seeing objects on the right, indicating oculomotor apraxia. Deep tendon reflexes were not found, and the upper and lower extremities muscle strength were grade III and grade II, respectively. The brain CT scan also revealed cerebellar atrophy.

(insert figure 2 here)

Fig 2. (A) Pedigree of patient #16's family and (B) Sanger sequencing results of patient #12 (II-2), her sibling (II-1) and her parents (I-1 and I-2) carried the compound heterozygotes of a known nonsense mutation c.8373C>A (p. Tyr2791Ter) in the *ATM* gene.

One patient (#4) had *ADK* gene mutation classified as VUS whose phenotypic features did not fully match the genotype. The patient was referred to our hospital at the age of 11 years with an intractable seizure with the onset of seizure at five years old and developmental regression of difficulty maintaining head position (truncal hypotonia). The physical examination revealed normocephalic, no facial dysmorphic, hypertonic, normal deep reflexes, and no pathological reflexes. The testing for serum methionine, S-adenosylmethionine (AdoMet), S-adenosylhomocysteine (AdoHcy), and homocysteine has not been commonly utilized in our

country. The *ADK* gene mutations are reported to have a phenotype of global developmental delay, seizures, dysmorphic face, and biochemical anomalies, such as hypermethioninemia with increased levels of AdoMet and AdoHcy, with normal homocysteine.

There were two patients with suggestive CNV. Patient #3, a boy with a heterozygous deletion finding, was identified at 15q11.2q13.1. Deletion at this genomic region is associated with autosomal dominant Prader-Willi syndrome and Angelman syndrome. The heterozygous deletion 15:(?_23684892)_(28544662_?) at 15q11.2q13.1 spans across 14 genes (*GOLGA6L2, MKRN3, MAGEL2, NDN, NPAP1, SNURF, SNRPN, UBE3A, ATP10A, GABRB3, GABRA5, GABRG3, OCA2,* and *HERC2*). He was born after 33 weeks of gestation along with his twin brother, and both were admitted to the Neonatal Incentive Care Unit (NICU) for 10 days due to neonatal asphyxia. This patient's twin brother died at the age of 10 months with intractable seizure and global developmental delay. The patient had developmental delay, ataxia of gait, speech impairment, apparent happy demeanor, microcephaly, and abnormal EEG. These clinical features were consistently found in patients with Angelman syndrome based on the consensus criteria by Williams et al. [14].

Patient #11, a boy with a heterozygous deletion of 6.1 Mb in length in the region of chromosome 1p31.3 leading to Chromosomal Deletion Syndrome 1p32-p31 (OMIM #613735). This region involves 49 genes including the *Nuclear Factor I/A (NFIA*) gene. The clinical findings of our patient have similar phenotypic features in several other cases of chromosome 1p32-p31 deletion syndrome such as ventriculomegaly, agenesis of the corpus callosum, facial dysmorphic, developmental delay, inguinal hernia, skeletal abnormalities, and urinary system defects. The phenotypic features of this syndrome vary depending on the size of the deletion region and the genes involved.

DISCUSSION

The introduction of WES into medicine has altered how physicians perform genetic etiology tracing in patients with suspected genetic disorders. WES has recently entered the field of clinical Mendelian disorder diagnosis, with success in diagnosing rare and heterogeneous genetic disorders. We aim to promote the WES examination as a diagnostic tool for pediatric patients with NGDs, especially those with unspecified clinical features. It decreases the time these patients must wait for a diagnosis, ends multi-year odysseys, influences their medical management, and optimizes genetic counseling for these families [15].

In our study, there were positive findings in 45% of patients (9 out of 20), and 13% had results suggestive of CNV. Overall, 10 patients (8 patients with pathogenic results and two patients with suggestive CNV) out of 20 patients had genetically and phenotypically consistent findings, while one patient with VUS had have an unrelated genotypic phenotype finding. A study in Saudi Arabia reported that 77% (20/26) of pediatric patients with various neurological disorders had positive WES results [16]. According to a French study, the rate of WES diagnosis reached 32.5% (14/43) in patients aged 2-40 years with intellectual disability (ID) and DEE [17]. A larger sample of 125 pediatric patients in a Dubai study revealed WES had a 40% diagnostic yield in patients with DEE, other focal and generalized epilepsy [18]. In a study of 2,000 children in the United States from different ethnic regions with nervous system dysfunction and developmental delay, WES was able to achieve a basic molecular diagnosis in 25.2% of instances, with 58% of these cases including new mutations [19]. Diagnostic yield for WES varies in studies around the world and is dependent on the size of the WES sample. The diagnostic yield of the WES results in this study, which used a small sample size, was comparable to that of other studies. However, this study still cannot describe the entire population.

The mean age of onset of NGDs in our study was 2 .45 ± 3.23 years, while in pediatric movement disorder patients in South Korea, it was 1.8 ± 3.0 years [7]. The mean age of genetic testing in our study was 6.57 ± 5.47 years, while in South Korea, it was 5.3 ± 5.2 years, revealing a delay of around 5 years between age of onset and genetic testing [7]. A study in Argentina even reported that the average time elapsed from the onset of symptoms to WES is 11 years [20]. The delay in genetic diagnosis occurs due to genetic and phenotypic heterogeneity of neurogenetic diseases that forces patients, families and even doctors to undertake a "diagnostic odyssey" [17,20]. In addition, the WES examination has only gained popularity in our country, Indonesia, during the past 5 years, making it difficult for us to conduct direct genetic testing on patients with atypical symptoms.

WES has a direct impact on the patient' therapy. Although sometimes specific therapy is not available for certain genetic defects but prevention of disease progression and future clinical features can be predicted. For, example, patient number #16 had a positive ATM mutation classified as pathogenic and diagnosed with ataxia telangiectasia (A-T) at nine years old. A-T is a multisystem disorder characterized by progressive neurological impairment, immunodeficiency, chromosomal instability, susceptibility to cancer, and ocular and cutaneous telangiectasia [21]. Evidence from the cerebellum of ATM-deficient mice suggests that oxidative stress may contribute to neuronal abnormalities in A-T. Antioxidant therapy for patients with A-T should be beneficial, given evidence of both diminished antioxidant capacity and elevated levels of oxidative stress in A-T cells [22]. Furthermore, there is also evidence that rehabilitation improves mobility, and balance in people with genetic degenerative ataxia [23]. This patient was given antioxidants (Vitamin A, B1, B2, B3, and B6) and a program of balance and strength exercises with a pediatric

physical therapist to prevent A-T progression. Our goal is to prevent rapid progression so that patients can continue to walk and live independently.

WES could reveal a rare illness variant that cannot be detected by conventional genetic tests. WES was used to diagnose Patient #14, a two-year-and-ten-month-old child with Angelman syndrome (AS) due to a point mutation in exon 9 of the *UBE3A* gene. This case report was already published [24]. AS is linked to mutations in the *UBE3A* gene, which is maternally inherited; however, in the case of our patient, the parents refused genetic testing. Several genetic tests, such as single nucleotide polymorphism arrays and methylation-specific multiplex ligation-dependent probe amplification (MS MLPA), are routinely used to screen patient with clinical suspicion of AS. However, these tests cannot detect point mutations; they can only detect maternal deletion, imprinting abnormalities, and paternal uniparental disomy (UPD). While, WES has recently been used to detect point mutation in the *UBE3A* gene [25]. However, WES also has limitations, such as requiring confirmation of other genetic examinations in certain cases. Patient #2, who is still associated with AS, the WES results of this patient showed suggestive CNV with a heterozygous deletion finding identified at 15q11.2q13.1. This case requires CMA or WGS genetic testing to confirm the findings suggestive of CNV.

In this small sample, it is possible to identify genes that cause rare neurogenetic disorders. Our patient #9, a 4 year old girl who was diagnosed with metachromatic leukodystrophy (MLD) (OMIM 250100), a sulfatide storage disease caused by deficient activity of the lysosomal enzyme arylsulfatase A (ARSA). On the basis of the age of symptom onset, there are three basic MLD phenotypes: late-infantile (< 30 months), juvenile (2.5–16 years), and adult (> 16 years). A severe phenotype is characterized by earlier onset of symptoms, accelerated disease progression, and reduced life expectancy. A search on Clinvar yielded only one finding with the same variant as

our patient (NM_000487.6:c.582dup; NP_000478.3:p.Trp195LeufsTer15) [26,27]. Our patient experienced a developmental regression at the age of 18 months, losing the ability to walk, speak, and grasp. The patient also had several generalized tonic-clonic seizures and is currently spastic in all four extremities. Neurologic examination showed positive signs of meningeal irritation and pathological reflexes (Oppenheim, Clonus and Babinski) and hypertonus. Head MRI examination shows extensive white matter changes in both hemisphere cerebri involving the splenium corpus callosum which leads to a leukodystrophy process. Beerepoot et al. reported patients with the same genotype but different phenotypes with 4 years of age of onset (early juvenile), with symptoms of peripheral neuropathy, ataxia and spasticity [27]. This can contribute to data on phenotypic heterogeneity which is expected to reduce the wandering diagnosis of similar patients in the future.

Although WES is comprehensive, its limitations should be recognized when negative findings are obtained, particularly when clinical indications are prominent; a multi-pronged approach is required before concluding the absence of genetic abnormalities. We summarized some recommendations when finding a negative WES result [17,28,29]. These included : 1) Add more detailed clinical phenotypic and family history data to make an accurate differential diagnosis.; 2) Perform an annual bioinformatic reanalysis of the data with a focus on uncommon variants that affect the coding sequence of genes listed in OMIM between analyses; 3) WES can consistently detect only single nucleotide variations and short (20bp) insertions and deletions in the protein-coding exonic regions of the nuclear genome. If large deletion/duplication, translocation, inversion, low-level mosaicism, or mitochondrial genome variants are suspected, it is advised to conduct tests specialized to detect these variant types.; 4) Testing (WES or Sanger sequencing) of biological parents and/or other family members is recommended to confirm the segregation of the variant(s).

Conclusions

WES is an important tool for diagnosing NGDs in children, especially those with unspecified clinical symptoms in routine practice. It decreases the time these patients must wait for a diagnosis, ends multi-year odysseys, gets personalized medicine therapy, and optimizes genetic counselling for these families.

Limitations

Given our limited sample size and sampling based on physicians' decisions, it is not possible to draw definite conclusions and generalizations. In the future, a comprehensive prospective study should be conducted to investigate the clinical characteristics that predict favorable genetic outcomes.

List of Abbreviations

ACMG: American College of Medical Genetics; AMP: Association for Molecular Pathology; CMA: chromosomal microarray; CNV: copy number variant; DEE: developmental and epileptic encephalopathy; EEG: Electroencephalogram; ENMG: Electroneuromyography; EMG: Electromyography; MS MLPA: methylation-specific multiplex ligation-dependent probe amplification ; MRI: Magnetic Resonance Imaging; NGDs: Neurogenetic disorders; NGS: Next-generation sequencing; SNV: single nucleotide variant; UPD: uniparental paternal disomy; VUS: Variant Uncertain Significance; WES: Whole-Exome Sequencing; WGS: Whole-Genome Sequencing

Declarations

Authors' contributions

All authors participated in the study's conception and design, data collection and analysis, article preparation and revision, and data collection and analysis. All authors have approved the publication of the current version and are responsible for all elements of the study.

Funding

This study was supported by grants from the Ministry of Research and Technology/National Research and Innovation Agency of the Republic of Indonesia to AT under contract numbers 018/E5/PG.02.00.PT/2022 and 1633/UN1/DITLIT/Dit-Lit/PT.01.00.2022 T. The funders had no role in study design, data collection and analysis, decision to publish, or preparation of the manuscript.

Competing interests

The authors state they have no competing interests.

Ethics approval and consent to participate

Patients older than 12 years old and/or their parents or guardian (for patients <12 years old) signed a written informed consent form to be included in this study. The Medical and Health Research Ethics Committee approved all recruitment and research protocol of the Faculty of Medicine, Public Health and Nursing, Universitas Gadjah Mada. Eligible patients signed an informed consent form (Ethical Clearance Number: K.E./0550/06/2020; Dated June 2020).

Availability of data and materials

The dataset used and/or analyzed during the current study are available from the corresponding author on reasonable request.

Acknowledgments

We would like to thank Gunadi, MD, PhD for his assistance in consulting several patient genetic results. We are grateful for the staff of the Office of Research and Publication, Faculty of Medicine, Public Health and Nursing, Universitas Gadjah Mada for English editing services and assistance in the proofreading and editing process.

Key messages

'What is Already Known?': Whole-Exome Sequencing has rapidly discovered a genetic basis in several neurogenetic diseases.

'What this Study Adds?': The diagnosis yield of WES in our study was 45 % (9/20). We identified nine positive results, including eight pathogenic single nucleotide variants in 8 genes, and one variant with uncertain significance in a single nucleotide variant.

Reference

[1] Karnebeek CDM van, Bowden K, Berry-Kravis E. Treatment of Neurogenetic Developmental Conditions: From 2016 into the Future. Pediatric Neurology 2016;65:1–13. https://doi.org/10.1016/j.pediatrneurol.2016.07.010.

[2] Good J-M, Atallah I, Castro Jimenez M, Benninger D, Kuntzer T, Superti-Furga A, et al. NGS-Based Diagnosis of Treatable Neurogenetic Disorders in Adults: Opportunities and Challenges. Genes (Basel) 2021;12:695. https://doi.org/10.3390/genes12050695.

[3] Mancuso M, Houlden H, Molnar MJ, Filla A, Breza M, Graessner H, et al. How to approach a neurogenetics diagnosis in different European countries: The European Academy of Neurology Neurogenetics Panel survey. Eur J Neurol 2022;29:1885–91. https://doi.org/10.1111/ene.15320.

[4] Fogel BL, Satya-Murti S, Cohen BH. Clinical exome sequencing in neurologic disease. Neurol Clin Pract 2016;6:164–76. https://doi.org/10.1212/CPJ.0000000000000239.

[5] Bargiela D, Yu-Wai-Man P, Keogh M, Horvath R, Chinnery PF. Prevalence of neurogenetic disorders in the North of England. Neurology 2015;85:1195–201. https://doi.org/10.1212/WNL.0000000000001995.

[6] Gonzaludo N, Belmont J, Gainullin V, Taft R. Estimating the burden and economic impact of pediatric genetic disease. Genetics in Medicine 2018;21. https://doi.org/10.1038/s41436-018-0398-5.
[7] Kim M-J, Yum M-S, Seo GH, Ko T-S, Lee BH. Phenotypic and Genetic Complexity in Pediatric Movement Disorders. Frontiers in Genetics 2022;13.
[8] Dunn P, Albury CL, Maksemous N, Benton MC, Sutherland HG, Smith RA, et al. Next Generation Sequencing Methods for Diagnosis of Epilepsy Syndromes. Front Genet 2018;9:20. https://doi.org/10.3389/fgene.2018.00020.
[9] Choi M, Scholl UI, Ji W, Liu T, Tikhonova IR, Zumbo P, et al. Genetic diagnosis by whole exome capture and massively parallel DNA sequencing. PNAS 2009;106:19096–101. https://doi.org/10.1073/pnas.0910672106.
[10] Grunseich C, Sarkar N, Lu J, Owen M, Schindler A, Calabresi PA, et al. Improving the efficacy of exome sequencing at a quaternary care referral centre: novel mutations, clinical presentations and diagnostic challenges in rare neurogenetic diseases. J Neurol Neurosurg Psychiatry 2021;92:1186–96. https://doi.org/10.1136/jnnp-2020-325437.
[11] Kalia SS, Adelman K, Bale SJ, Chung WK, Eng C, Evans JP, et al. Recommendations for reporting of secondary findings in clinical exome and genome sequencing, 2016 update (ACMG SF v2.0): a policy statement of the American College of Medical Genetics and Genomics. Genetics in Medicine 2017;19:249–55. https://doi.org/10.1038/gim.2016.190.
[12] Seo GH, Kim T, Choi IH, Park J-Y, Lee J, Kim S, et al. Diagnostic yield and clinical utility of whole exome sequencing using an automated variant prioritization system, EVIDENCE. Clin Genet 2020;98:562–70. https://doi.org/10.1111/cge.13848.
[13] Richards S, Aziz N, Bale S, Bick D, Das S, Gastier-Foster J, et al. Standards and guidelines for the interpretation of sequence variants: a joint consensus recommendation of the American College of Medical Genetics and Genomics and the Association for Molecular Pathology. Genet Med 2015;17:405–23. https://doi.org/10.1038/gim.2015.30.
[14] Williams CA, Beaudet AL, Clayton-Smith J, Knoll JH, Kyllerman M, Laan LA, et al. Angelman syndrome 2005: Updated consensus for diagnostic criteria. Am J Med Genet 2006;140A:413–8. https://doi.org/10.1002/ajmg.a.31074.
[15] Whole exome sequencing in neurogenetic odysseys: An effective, cost- and time-saving diagnostic approach | PLOS ONE n.d. https://journals.plos.org/plosone/article?id=10.1371/journal.pone.0191228 (accessed October 29, 2022).
[16] Muthaffar O. The utility of whole exome sequencing in diagnosing pediatric neurological disorders. Balkan Journal of Medical Genetics 2021;23:17–24. https://doi.org/10.2478/bjmg-2020-0028.
[17] Thevenon J, Duffourd Y, Masurel-Paulet A, Lefebvre M, Feillet F, El Chehadeh-Djebbar S, et al. Diagnostic odyssey in severe neurodevelopmental disorders: toward clinical whole-exome sequencing as a first-line diagnostic test. Clin Genet 2016;89:700–7. https://doi.org/10.1111/cge.12732.
[18] Rochtus A, Olson HE, Smith L, Keith LG, El Achkar C, Taylor A, et al. Genetic diagnoses in epilepsy: The impact of dynamic exome analysis in a pediatric cohort. Epilepsia 2020;61:249–58. https://doi.org/10.1111/epi.16427.

[19] Yang Y, Muzny DM, Xia F, Niu Z, Person R, Ding Y, et al. Molecular Findings Among Patients Referred for Clinical Whole-Exome Sequencing. JAMA 2014;312:1870–9. https://doi.org/10.1001/jama.2014.14601.
[20] Córdoba M, Rodriguez-Quiroga SA, Vega PA, Salinas V, Perez-Maturo J, Amartino H, et al. Whole exome sequencing in neurogenetic odysseys: An effective, cost- and time-saving diagnostic approach. PLoS One 2018;13:e0191228. https://doi.org/10.1371/journal.pone.0191228.
[21] Rothblum-Oviatt C, Wright J, Lefton-Greif MA, McGrath-Morrow SA, Crawford TO, Lederman HM. Ataxia telangiectasia: a review. Orphanet J Rare Dis 2016;11:159. https://doi.org/10.1186/s13023-016-0543-7.
[22] Lee S-A, Lee K-M, Lee S-J, Yoo K-Y, Park S, Noh D-Y, et al. Antioxidant Vitamins Intake, Ataxia Telangiectasia Mutated (ATM) Genetic Polymorphisms, and Breast Cancer Risk. Nutrition and Cancer 2010;62:1087–94. https://doi.org/10.1080/01635581.2010.492088.
[23] Unes S, Tuncdemir M, Eroglu-Ertugrul NG, Kerem Gunel M. Effectiveness of Physical Therapy on Ataxia-Telangiectasia: A Case Report. Pediatr Phys Ther 2021;33:E103–7. https://doi.org/10.1097/PEP.0000000000000813.
[24] Triono A, Iskandar K, Nugrahanto AP, Hadiyanto ML, Gunadi, Herini ES. The role of whole exome sequencing in the UBE3A point mutation of Angelman Syndrome: A case report. Annals of Medicine and Surgery 2022;73:103170. https://doi.org/10.1016/j.amsu.2021.103170.
[25] Li H, Yang H, Lv N, Ma C, Li J, Shang Q. Whole exome sequencing and methylation‑specific multiplex ligation‑dependent probe amplification applied to identify Angelman syndrome due to paternal uniparental disomy in two unrelated patients. Mol Med Report 2019. https://doi.org/10.3892/mmr.2019.10339.
[26] VCV000973783.1 - ClinVar - NCBI n.d. https://www.ncbi.nlm.nih.gov/clinvar/variation/973783/?oq=%22NM_000487.6:c.582dup%22%5Bvarname%5D&m=NM_000487.6(ARSA):c.582dup%20(p.Trp195fs) (accessed January 29, 2023).
[27] Beerepoot S, van Dooren SJM, Salomons GS, Boelens JJ, Jacobs EH, van der Knaap MS, et al. Metachromatic leukodystrophy genotypes in The Netherlands reveal novel pathogenic ARSA variants in non-Caucasian patients. Neurogenetics 2020;21:289–99. https://doi.org/10.1007/s10048-020-00621-6.
[28] Seo GH, Lee H, Lee J, Han H, Cho YK, Kim M, et al. Diagnostic performance of automated, streamlined, daily updated exome analysis in patients with neurodevelopmental delay. Molecular Medicine 2022;28:38. https://doi.org/10.1186/s10020-022-00464-x.
[29] Pena L, Jiang Y, Schoch K, Spillmann R, Walley N, Stong N, et al. Looking beyond the exome: A phenotype-first approach to molecular diagnostic resolution in rare and undiagnosed diseases. Genetics in Medicine : Official Journal of the American College of Medical Genetics 2017;20. https://doi.org/10.1038/gim.2017.128.

Figure 1

Click here to access/download;Figure;Fig 1.tiff

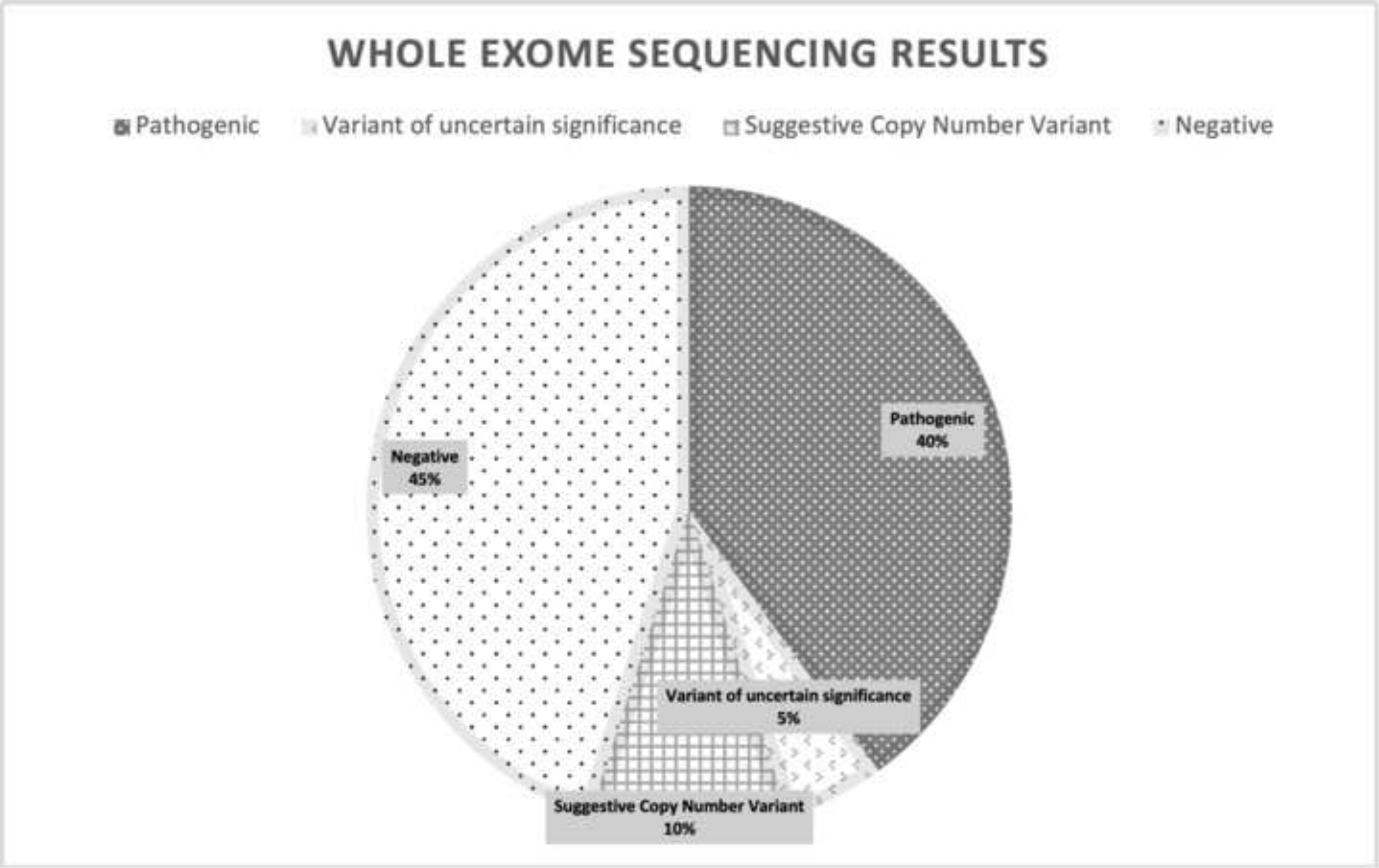

Figure 2

Click here to access/download;Figure;Fig 2.tiff

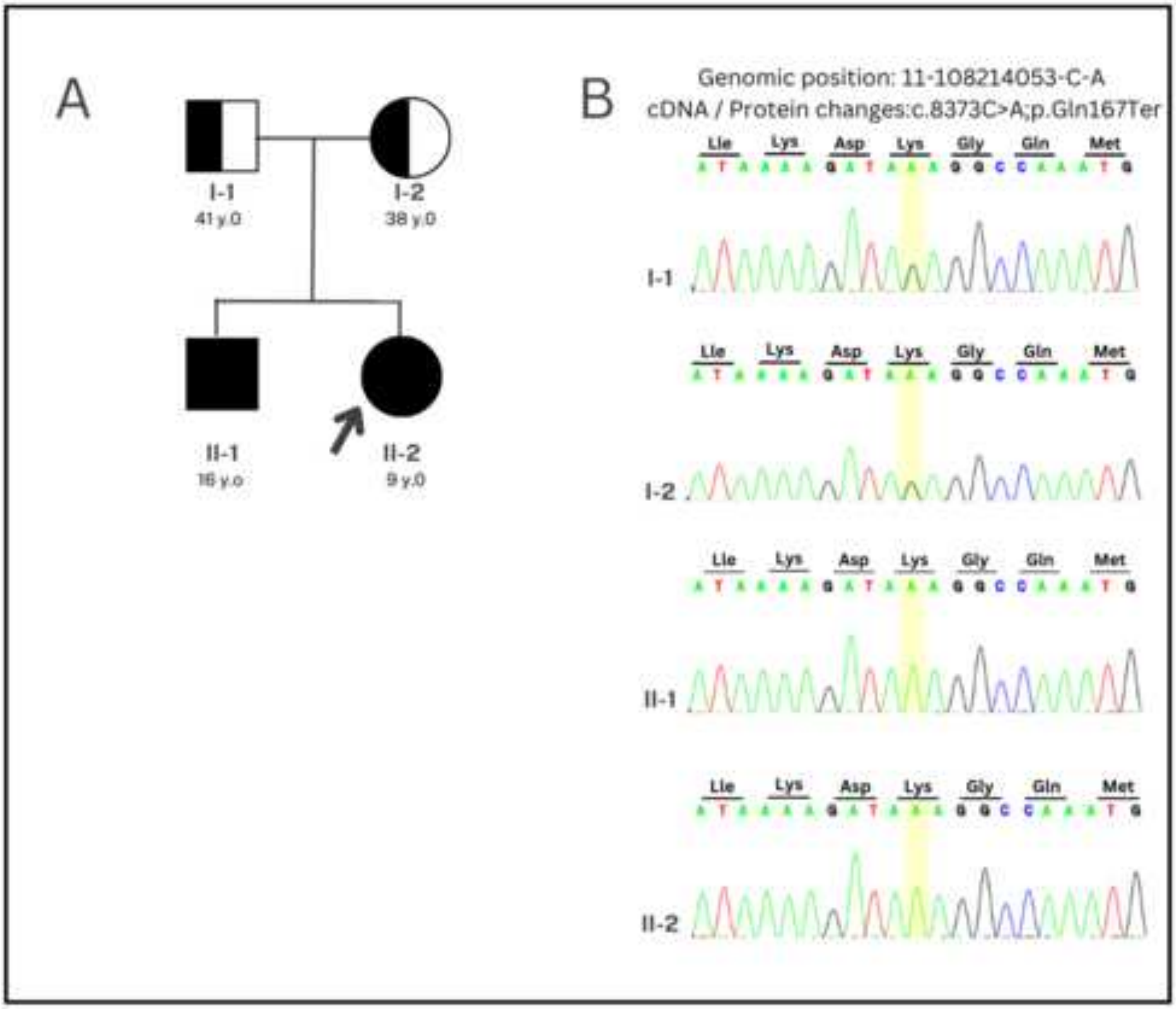

ethical approval letter

Click here to access/download
Supporting Information
ethical approval letter SYNAPS.pdf
